# High-Density Dielectrophoretic Microwell Array for Detection, Capture, and Single-Cell Analysis of Rare Tumor Cells in Peripheral Blood

**DOI:** 10.1371/journal.pone.0130418

**Published:** 2015-06-24

**Authors:** Atsushi Morimoto, Toshifumi Mogami, Masaru Watanabe, Kazuki Iijima, Yasuyuki Akiyama, Koji Katayama, Toru Futami, Nobuyuki Yamamoto, Takeshi Sawada, Fumiaki Koizumi, Yasuhiro Koh

**Affiliations:** 1 Life Science Research Laboratory, Tosoh Corporation, Kanagawa, Japan; 2 Third Department of Internal Medicine, Wakayama Medical University, Wakayama, Japan; 3 Shien-Lab, National Cancer Center Hospital, Tokyo, Japan; Queen's University at Kingston, CANADA

## Abstract

Development of a reliable platform and workflow to detect and capture a small number of mutation-bearing circulating tumor cells (CTCs) from a blood sample is necessary for the development of noninvasive cancer diagnosis. In this preclinical study, we aimed to develop a capture system for molecular characterization of single CTCs based on high-density dielectrophoretic microwell array technology. Spike-in experiments using lung cancer cell lines were conducted. The microwell array was used to capture spiked cancer cells, and captured single cells were subjected to whole genome amplification followed by sequencing. A high detection rate (70.2%–90.0%) and excellent linear performance (R^2^ = 0.8189–0.9999) were noted between the observed and expected numbers of tumor cells. The detection rate was markedly higher than that obtained using the CellSearch system in a blinded manner, suggesting the superior sensitivity of our system in detecting EpCAM− tumor cells. Isolation of single captured tumor cells, followed by detection of *EGFR* mutations, was achieved using Sanger sequencing. Using a microwell array, we established an efficient and convenient platform for the capture and characterization of single CTCs. The results of a proof-of-principle preclinical study indicated that this platform has potential for the molecular characterization of captured CTCs from patients.

## Introduction

Molecular approaches to improving cancer therapy efficacy are increasing in number and sophistication, creating a need for companion diagnostics to determine therapeutic strategies. Particular actionable genomic aberrations have been shown to enable prediction of response to molecularly targeted treatments [1]. Conventionally, this strategy relies on analysis of primary tumor samples; thus, there is an urgent need for minimal invasiveness and greater accessibility [2]. Circulating tumor cells (CTCs) offer an alternative source for the detection of genetic alterations, as a form of “liquid biopsy” [3–7]. CTCs, tumor cells shed from the primary tumor, which circulate in the blood stream, are found in the peripheral blood of patients with metastatic cancer. Currently, the CellSearch system is the only FDA-approved CTC enumeration system. Through the use of this system, baseline and follow-up CTC levels have been reported to be strong predictors of progression-free and overall survival in monitoring patients with metastatic breast, prostate, and colorectal cancer [8]. The clinical significance of CTCs has also been evaluated in patients with non-small cell lung, small cell lung, and gastric cancers [9–12].

To date, a variety of platforms capable of enriching and detecting CTCs have been developed [5]. They are generally categorized as anti-epithelial cell adhesion molecule (EpCAM) antibody-coated isolation systems, as represented by the CellSearch system [13–15]; anti-EpCAM antibody independent systems [16–18]; or membrane filtration systems [19–21]. Molecular characterization studies have revealed, however, that CTCs are highly heterogeneous, a finding that emphasizes the need for single-cell approaches. As a means of understanding hematogenous tumor cell dissemination in cancer progression, the molecular characterization of CTCs at a single-cell level remains technically challenging. Various studies addressing this issue have been under development and evaluation [22–26]. The identification and characterization of single CTCs typically involve a combination of complex enrichment and single-cell isolation procedures (e.g., CellSearch followed by micromanipulation or FACS; Refs. 24 and 26). Dielectrophoretic technology has been used in the isolation and molecular characterization of single tumor cells, including CTCs [27–30]. In these earlier studies, cell loss during sample handling between enrichment and isolation is a critical concern in the case of rare-CTC cohorts [24, 26, 27].

In this study, we introduced a new approach for the isolation of single CTCs detected and captured by a newly developed dielectrophoretic device. This novel device enables the detection and single-cell isolation of rare tumor cells for subsequent molecular analysis. Here we report the results of a proof-of-principle preclinical study based on a novel workflow comprising negative enrichment and image-based immunophenotypic analysis using a fluorescence microscope, followed by mutation analysis of the isolated single tumor cells. This is an efficient and convenient platform based on a dielectrophoretic system, and promising preclinical results were obtained for possible future clinical application.

## Material and Methods

### Ethical statement

The study protocol was approved by the Institutional Review Board of the Life Science Research Laboratory, Tosoh Corporation (Protocol TR14-01). Written informed consent was obtained from all participating donors prior to sample collection.

### Buffer, reagents, and antibodies

For enrichment of mononucleated cells from blood, Lymphoprep density gradient media (Density: 1.077 ± 0.001 g/ml, Osmolality: 280 ± 15 mOsm/kg-H_2_O, Axis-Shield, Oslo, Norway) was used. For depletion of white blood cells, RosetteSep reagent Human CD45 Depletion Cocktail was used. Briefly, the RosetteSep reagent crosslinks multiple red blood cells and unwanted cells (e.g., white blood cells) to increase the density of the unwanted cells. For washing the enriched mononucleated cells, Phosphate-Buffered Saline (PBS, purchased from Wako Pure Chemical Industries, Osaka, Japan) containing 0.35% trisodium citrate was used. A lysing solution, containing 9.0 g/L of NH_4_Cl, 1.0 g/L of KHCO_3_, and 0.037 g/L of EDTA-4Na, was used for red blood cell lysis. A 300 mM mannitol solution (Sigma Aldrich, St. Louis, MO) was used as a solution with suitable conductivity for dielectrophoresis and appropriate osmotic pressure to allow living cells to maintain their shape. A BSA solution, consisting of PBS containing 0.1% BSA (Sigma Aldrich), was used for pre-coating BSA on the surface of the microwell array substrates. A 300 mM mannitol solution containing poly-L-lysine was used for the cell attachment procedure. The chamber-washing solution consisted of PBS containing 1% BSA and 0.05% Polyoxyethylene(20) Sorbitan Monolaurate (Tween20) (Wako Pure Chemical Industries). The fluorescein isothiocyanate (FITC)-conjugated anti-CK monoclonal Antibody (mAb) CK3-6H5 (mouse IgG_1_), unlabeled anti-CK mAb CK3-6H5 (mouse IgG_1_), and phycoerythrin (PE)-conjugated anti-CD45 mAb 5B1 (mouse IgG_2a_), and the FcR blocking reagent, were purchased from Milenyi Biotec (Bergisch-Gladbach, Germany). The Alexa Fluor 488 conjugated Anti-Pan-CK mAb AE1/AE3 (mouse IgG_1_), unlabeled anti-Acidic-CK mAb AE1 (mouse IgG_1_), and unlabeled anti-Basic-CK mAb AE3 (mouse IgG_1_) were purchased from Affymetrix (Santa Clara, CA). The PE-conjugated anti-CD45 mAb J33 (mouse IgG_1_) was purchased from Beckman Coulter (Marseille, France). The Alexa Fluor 488 conjugated goat anti-mouse IgG_1_ isotype specific antibody, and the Calcein Blue AM reagent were purchased from Life Technologies (Carlsbad, CA). 4',6-Diamidino-2-phenylindole dihydrochloride (DAPI) solution was purchased from DOJINDO Laboratories, Kumamoto, Japan.

### Cell culture

The cell line SK-BR-3, PC-9, PC-14, H69, SBC-3 and H1975 were used in this work. Details of the cell line and the cell culture can be found in the Supporting Information.

### Theoretical and experimental description of dielectrophoresis

Dielectrophoresis (DEP) refers to the process whereby dielectric particles, for example living cells, in a non-uniform electrical field, undergo a force (DEP force). The DEP force $\vec{F}$, which a non-uniform electric field $\vec{E}$ exerts on a spherical cell in a fluid medium, is expressed as:

$$\vec{F}=2\pi \varepsilon_{m}a^{3}\frac{\varepsilon_{cell}-\varepsilon_{m}}{\varepsilon_{cell}+2\varepsilon_{m}}\vec{\nabla }\left|{\vec{E}}^{2}\right|\;,$$

where *ε*_*m*_ and *ε*_*cell*_ are the dielectric permittivity of the medium and the cell, respectively, and *a* is the particle radius [31]. Thus, the DEP force exerted on a cell is proportional to the volume of the cell, the difference between the relative permittivity of the cell and the surrounding medium, and the gradient of the square of the electric field. When *ε*_*cell*_ − *ε*_*m*_ is greater than 0, the cell is pulled toward the region of strong field.

The real DEP force exerted upon the cell is also dependent on the frequency of the applied electric field, and on the conductivity of the surrounding fluid medium. This is because the elements of dielectric permittivity, *ε*_*m*_ and *ε*_*cell*_, are dependent on the frequency of the field and the conductivity of the medium. Medium conductivity of < 200 μS/cm is preferable, in order to prevent excess current flow in the medium; and AC voltage with a square wave is preferable to that with a sine wave, for rapid entrapment of cells.

**Fig 1:**
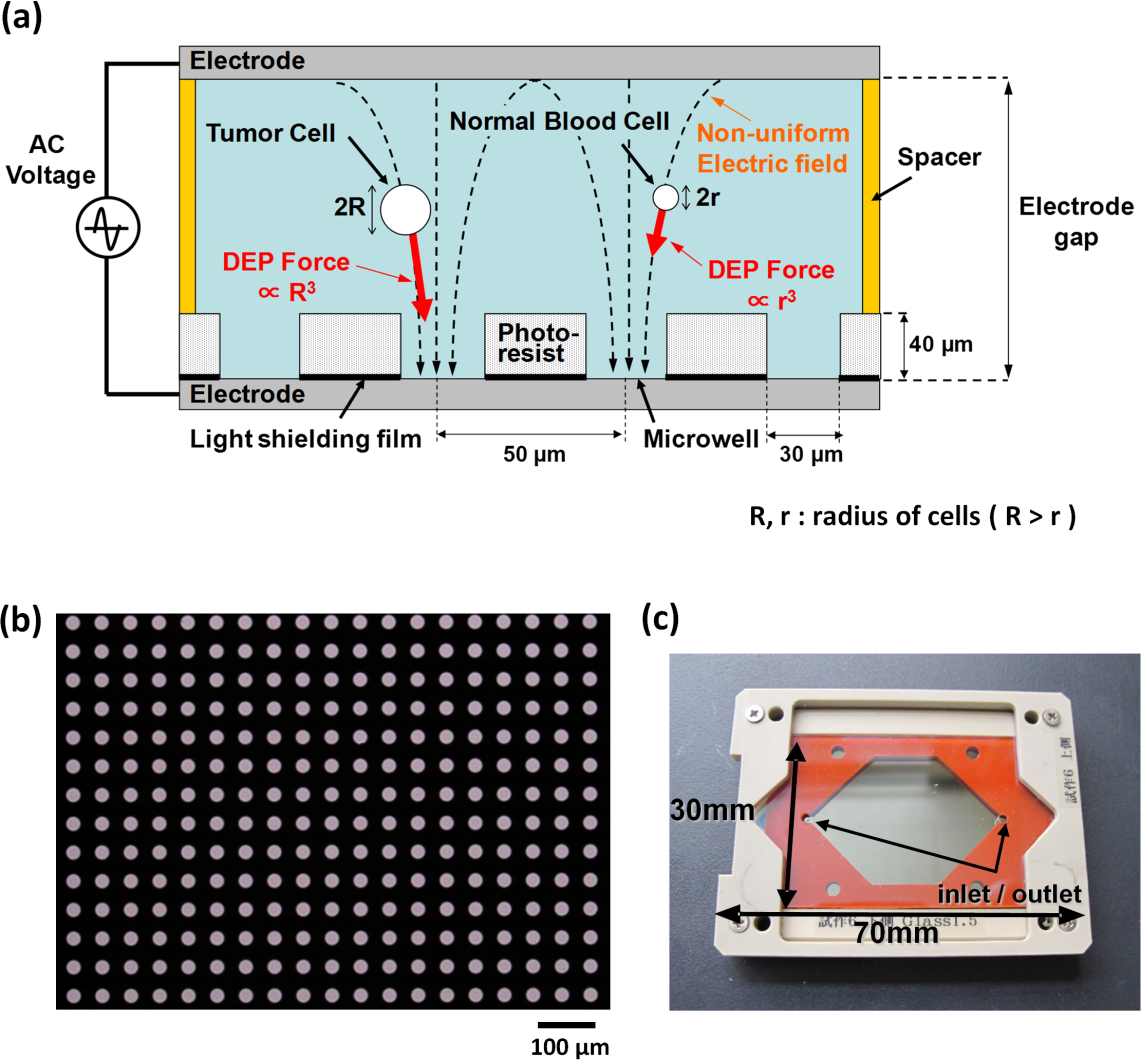
Features of the Cell Entrapment Chamber Utilizing Dielectrophoresis (DEP). (a) The suspension containing target cells is introduced into the space between the upper and lower electrode substrates. The electric field generated between the upper electrode and the lower electrode exposed on the bottom surface of each microwell shows non-uniformity in this configuration, with the result that a DEP force is exerted on the cells. The DEP force exerted on a cell is proportional to the volume of the cell, as noted in the Material and Methods section; thus, relatively larger CTCs (radius = R) are preferentially entrapped into the microwells, compared with smaller white blood cells (radius = r). (b) The microwell array fabricated on the plane ITO electrode. The center-to-center distance between microwells is 50 μm. The Cr light-shielding film is sheeted beneath the entire area of the photoresist, with the exception of the microwell regions. (c) The cell entrapment chamber, consisting of the microwell array substrate and another glass substrate coated with ITO thin film, with an open space between them which includes a silicon rubber sheet spacer of thickness 1 mm (thus the distance between the pair of electrodes is 1 mm), and with roughly 300,000 microwells opening onto the space, enables the accommodation of roughly 800 μL of cell-containing suspension in the open space.

Fig 1A shows a schematic image of the entrapment of cells in a microwell array, utilizing the DEP force. The DEP force exerted on a cell is proportional to the volume of the cell, as mentioned above; thus, relatively larger CTCs, as compared with smaller white blood cells, are preferentially entrapped in such microwells. As shown in Fig 1B, hundreds of thousands of micropores are patterned on a photoresist (SU-8) film, sheeted on a glass substrate (length 70 mm × breadth 40 mm × thickness 1 mm) coated with an indium tin oxide (ITO) thin-film electrode (thickness 150 nm, resistivity 10 Ω/sq). Thus, the ITO electrode is exposed on the bottom surface of each well. Each well has a 30 μm diameter, 40 μm depth, and 50 μm interval. In addition, light-shielding Cr thin film is sheeted between the ITO electrode and the photoresist film, covering the entire ITO surface, with the exception of the regions corresponding to the respective microwells. Fig 1C shows the cell entrapment chamber, which consists of the microwell array substrate and another glass substrate coated with ITO thin film, with an open space between them which includes a silicon rubber sheet spacer of thickness 1 mm (thus the distance between the pair of electrodes is 1 mm), with roughly 300,000 of microwell opened to the space, enables the accommodation of roughly 800 μL of suspension containing cells in the open space. Since the SU-8 photoresist is a good insulation material, the electric field generated between the upper electrode and the lower electrode exposed on the bottom surface of each microwell shows non-uniformity in this configuration, and therefore a DEP force is exerted on the cells. When AC voltage is applied between the pair of electrodes, with a frequency of 100 kHz-10 MHz and electric potential of 20–50 Vp-p, a non-uniform electric field with intensity of 40–100 kV/m is generated in each microwell in the above configuration, resulting in a typical living human cell of diameter 5–30 μm being trapped in each microwell within a few minutes.

### Fabrication of microwell array substrate

S1 Fig shows a schematic diagram of the photolithographic method employed, with corresponding etching, to fabricate the microwell array substrate. Further information of fabrication of the microwell array substrate can be found in the Supporting Information. The fabricated microwell array substrate was soaked overnight in PBS containing 0.1% BSA, to make its surface hydrophilic, and then taken out, washed briefly with pure water, and dried immediately before the experiment.

### Negative enrichment of tumor cells

Blood samples were voluntarily donated by healthy employees of Tosoh Corporation on the day of the experiments, and collected in Venoject II vacuum collection tubes supplemented with EDTA-2K (TERUMO, Tokyo, Japan).

In the spike-in experiments, 3.0 mL samples of blood were spiked with tumor cells, whose number was adjusted in advance by means of fluorescently labeling with Calcein Blue AM reagent to distinguish live from dead cells. Negative enrichment of the tumor cells was performed using the RosetteSep reagent Human CD45 Depletion Cocktail (STEMCELL technologies, Vancouver, BC, Canada) according to the manufacturer’s protocol with some modification. Further information of procedure of negative enrichment of tumor cells can be found in the Supporting Information. As a result of the procedure, a suspension of tumor cell-enriched mononucleated cells with conductivity of < 200 μS/cm was obtained. The resulting number of mononucleated cells ranged from 1 ×10^5^ to 5 × 10^5^, depending on the donor’s blood condition.

### Entrapment of cells in the microwell array

The suspension of tumor cell-enriched mononucleated cells was then loaded into the cell entrapment chamber (described above), followed by immediate application of 20 Vp-p AC voltage, with a square-wave shape and frequency of 3 MHz, by a function generator (WF1974, NF Corporation, Tokyo, Japan) for 3 minutes, so as to entrap cells in the microwells using the DEP force. Then, poly-L-lysine solution was introduced into the chamber with the DEP force being applied continuously, followed by incubation for 3 min at RT, to attach the cells to the bottom of the wells. After removal of the poly-L-lysine solution, 50%(v/v) ethanol supplemented with 1%(v/v) HCHO (formaldehyde) was introduced into the chamber, followed by incubation for 10 min at RT, for permeabilization and fixation. After removal of the ethanol solution, the chamber was washed with the chamber-washing solution, followed by introduction of the chamber-washing solution supplemented with 1:10 diluted FcR blocking reagent and incubation at RT for 10 min.

### Immunofluorescent staining and capture of images

For the cell lines which displayed high CK expression (SK-BR-3 and PC-9), a PBS-based immunofluorescent staining solution containing 1% BSA, 0.05% Tween20, 1:16 diluted CK-FITC (CK3-6H5), 1 μg/mL of AE1/AE3-Alexa Fluor 488, 0.5 μg/mL of DAPI, 1:8 diluted CD45-PE (J33), and 1:10 diluted FcR blocking reagent was introduced into the chamber, followed by incubation for 30 min at RT. After removal of unbounded antibodies, the chamber was again washed three times with the chamber-washing solution, and then filled with 300 mM mannitol solution.

For the cell lines which displayed low CK expression (PC-14, H69 and SBC-3), a PBS-based immunofluorescent staining solution containing 1% BSA, 0.05% Tween20, 5 μg/mL of CK3-6H5, AE1, AE3 anti-CK mAbs, and 1:10 diluted FcR blocking reagent was introduced into the chamber, followed by incubation for 30 min. After removal of the unbound antibodies and three-fold washing with the chamber-washing solution, a secondary antibody solution containing 1% BSA, 0.05% Tween20, 10 μg/mL of anti-mouse IgG_1_- Alexa Fluor 488, 0.5 μg/mL of DAPI, 1:11 diluted CD45-PE (5B1), and 1:10 diluted FcR blocking reagent was introduced, followed by incubation for 20 min at RT. After removal of the unbounded antibodies, the chamber was again washed three times with the chamber-washing solution, and then filled with 300 mM mannitol solution.

After immunofluorescent staining, the chamber was mounted on the x-y translational stage located on an IX 71 fluorescence microscope (Olympus, Tokyo, Japan). Fluorescent images of the respective wavelengths (for DAPI, FITC, and PE) of the entire area of the microwell array were captured with an ADT-100 EM-CCD camera (FLOVEL, Tokyo, Japan). A 4× objective lens was used for the initial image capture, and a 10× objective lens was used for secondary image capture for detailed analysis. Image capture and analysis were performed with custom-made software.

### Spiking experiment

Cancer cell lines (SK-BR-3, PC-9, PC-14, H69, and SBC-3) were cultured and harvested with trypsin-EDTA (except for the floating cell line H69) immediately before being spiked into 3 mL of blood from a healthy donor. The blood sample, spiked with number-adjusted cancer cells, was immediately treated with the serial procedures described above. The number of spiked cancer cells were divided into 3 sets: around 10 cells (8–13 cells, actually counted before being spiked-in), around 100 cells (80–114 cells, actually counted before being spiked-in), and around 1000 cells (approximate number of cells). The experiments were performed two to three times for each cell-number set (the detailed number of replicates can be seen in S2 Table). The detection rate and coefficient of variation (CV) were calculated for each experiment, except in the case of the ~1000-cell set. Plots of the number of detected cells against the number of spiked cells were fitted by linear fitting, and slopes and determination coefficients (*R* ^2^) were calculated. To evaluate the efficacy of the system in the case of an exceptionally low number of cancer cells, spiking experiments involving a spiked-cell number of up to 5 cells were conducted twice each for two different cell lines (SK-BR-3 and PC-9).

### Isolation of single tumor cells by micromanipulation

Tumor cells, as defined above, were isolated from the microwell by a micromanipulated aspirator consisting of a syringe pump equipped with a custom-made L-shaped glass capillary with an inner tip diameter of 30 μm. Single aspirated tumor cells were released into 2 μL of 300 mM mannitol solution in respective PCR tubes.

### Detection of mutation in tumor specific genes from single tumor cells.

For sequencing analysis of genomic DNA from the isolated single tumor cells, whole-genome amplification (WGA) was performed with the *Ampli*1 WGA Kit (Silicon Biosystems, Bologna, Italy) according to the manufacturer’s protocol. After confirmation of the success of the WGA by gel electrophoresis, ~100 ng of WGA products were used for Sanger direct sequencing of *epidermal growth factor receptor* (*EGFR*).

## Results

### Assay development for rare cell entrapment after negative depletion enrichment

Entrapment of cells in the microwell array utilizing dielectrophoresis (DEP) was demonstrated (S1 Video). Theoretically, the amplitude of the DEP force exerted on a particle is linear to the cube of the particle radius [31, 32], and relatively larger cells were experimentally observed to be drawn into the microwells more rapidly than relatively smaller cells (S1 Video). Since the amplitude of the DEP force is also linear to the difference between the relative permittivity of the cell and the surrounding medium, the DEP force exerted on cells with partially damaged cell membranes, allowing unfavorable cytosolic fluid exchange with the surrounding medium, is relatively lower than that exerted on cells with intact cell membranes. Thus, inter-cellular differences, not only in cell size but in membrane damage status, are conjectured to be a cause of differences in the velocity of cell locomotion. We also confirmed that the frequency of AC voltage applied between the pair of electrodes influences cell entrapment efficacy (i.e., the number of entrapped cells per total number of cells introduced into the cell entrapment chamber). For live cells, the efficacy of entrapment was 100% with a frequency of 100 kHz, 1 MHz, or 3 MHz, and slightly lower (98%) with 10 MHz, AC voltage (S4 Fig). Because there was a fear of live cell damage arising from stress induced by the applied electric field, with a frequency of 100 kHz to 1 MHz (maximum at 200 kHz) for cancer cells and with a frequency of 1 MHz to 2 MHz for lymphocytes [33, 34], we applied AC voltage with a frequency of 3 MHz in this study.

### Evaluation of the efficacy of the assay protocol and the system through spiking experiments

**Fig 2:**
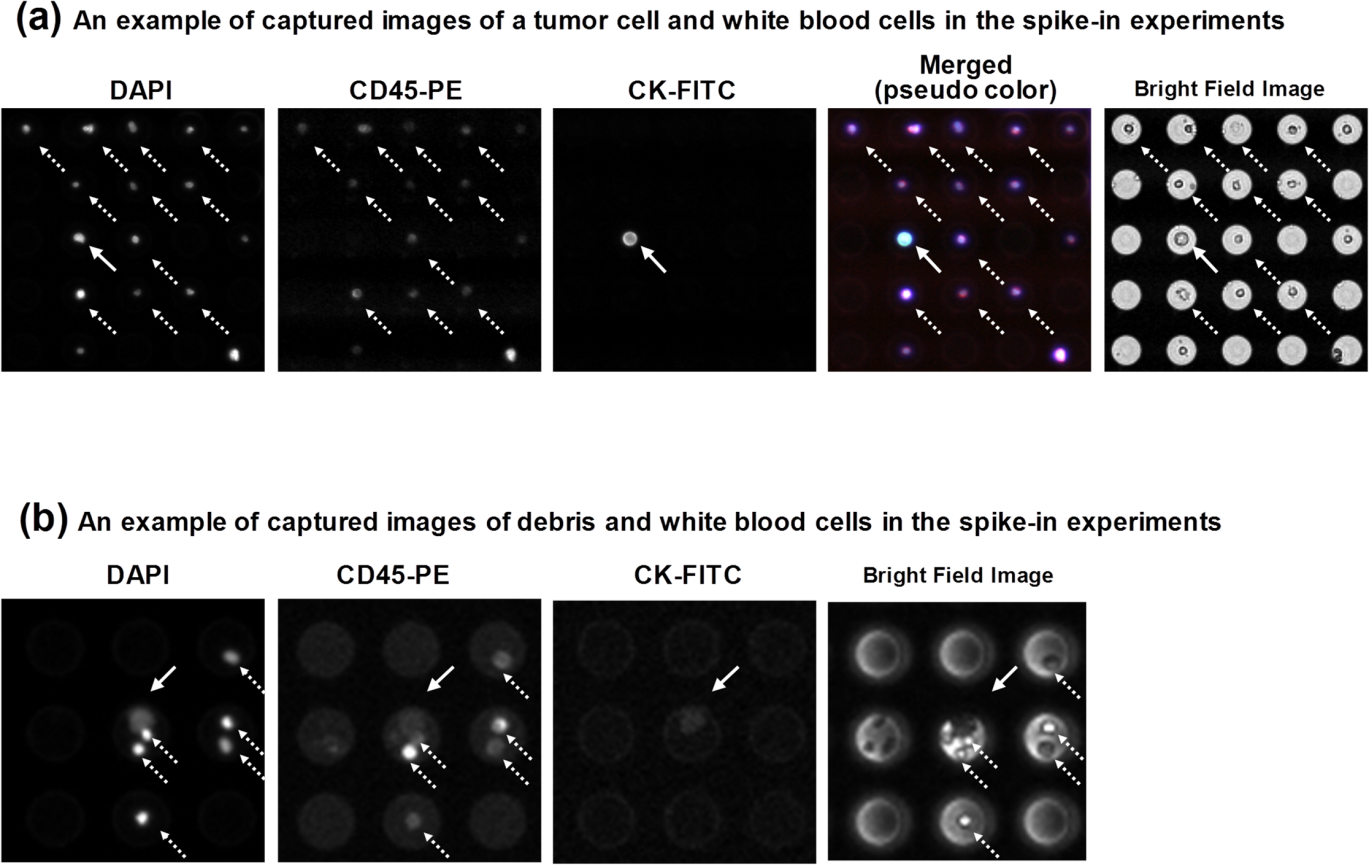
Typical Examples of Captured Images of Tumor Cells and White Blood Cells in the Spike-in Experiments. Pictured are tumor cell-enriched mononucleated cells after immunofluorescent staining with DAPI, CK-FITC, or CD45-PE. Fluorescent images of the respective wavelengths (for DAPI, FITC, and PE) over the entire area of the microwell array were captured. Tumor cells were defined according to the criteria DAPI+ and CK+ and CD45–, while the white blood cells were defined as DAPI+ and CK–and CD45+. (a) An example of captured images of a tumor cell and white blood cells in the spike-in experiments. The SK-BR-3 cell line was spiked into blood, followed by serial procedures. The cell indicated by the solid arrow was defined as a tumor cell, while those indicated by dotted arrows were defined as white blood cells. The images were captured with a 10× objective lens. (b) An example of captured images of debris and white blood cells in the spike-in experiments. Bright-field image cells with black filling (solid arrow) were defined as debris, while the dotted-arrowed cells were defined as white blood cells. The images were captured with a 4× objective lens.

**Fig 3:**
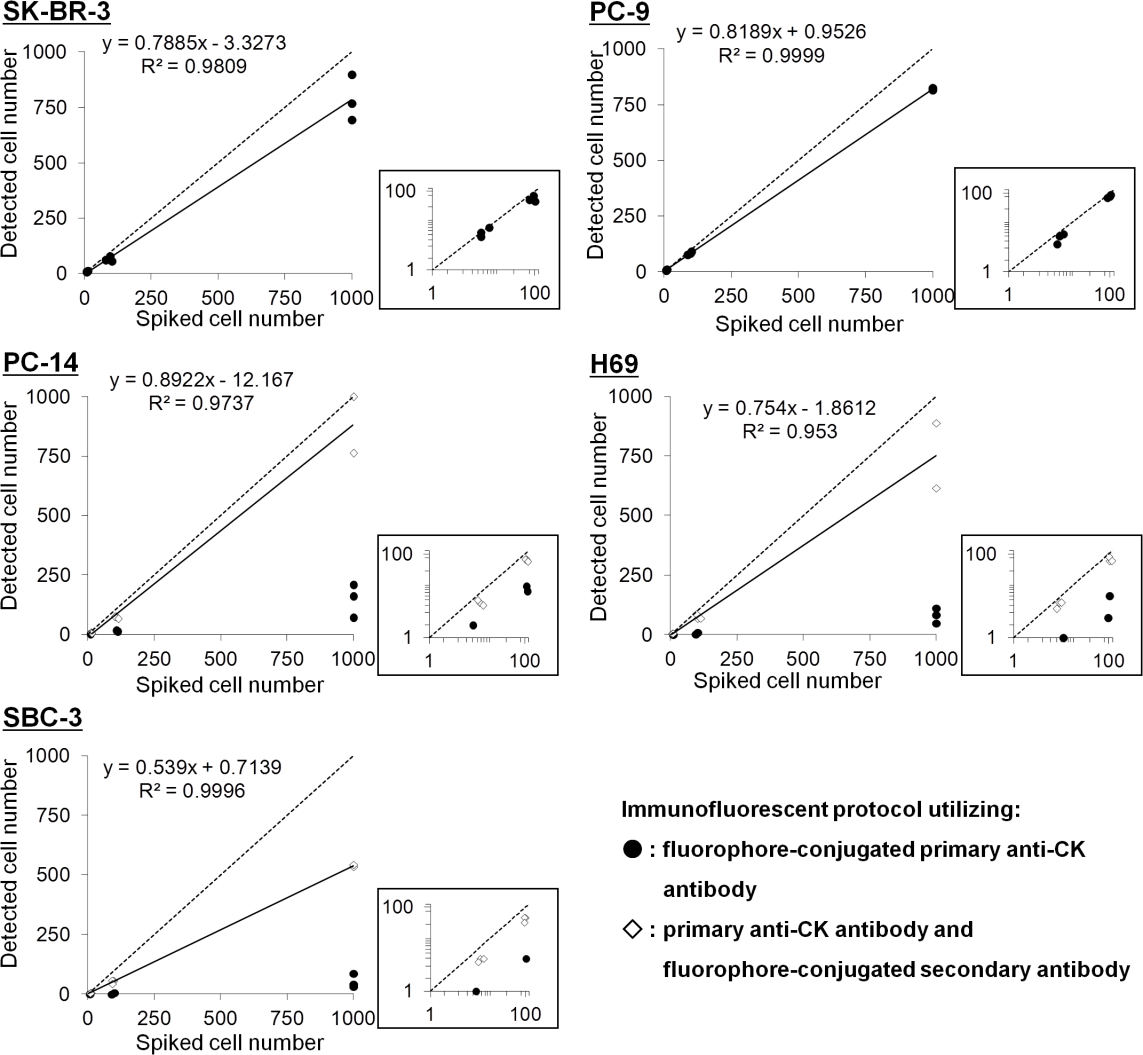
Plots of the Number of Detected Cells against the Number of Spiked Cells in the Spike-in Experiments. Cultured tumor cells (SK-BR-3 (breast), PC-9 (non-small cell lung), PC-14 (non-small cell lung), H69 (small cell lung), and SBC-3 (small cell lung)) were separately spiked, in numbers of 10, 100, and 1000, into 3 mL of blood obtained from healthy donors. After enrichment of mononucleated cells from the blood, the cells were entrapped in microwells, followed by immunofluorescent staining with DAPI, chromophore conjugated anti-CK mAb, and anti-CD45 mAb. Then, fluorescent images of the entire area of the microwell array were captured, and the numbers of tumor cells were measured (solid circles). For PC-14, H69, and SBC-3, a further staining protocol, using Alexa Fluor 488 conjugated secondary antibody, was applied for amplification of the weak CK signal (diamonds). The circles and diamonds represent individual data points. The straight lines are the linear fittings, with their slopes and correlation coefficients (R^2^) given on the plots. Dotted lines represent the function y = x. The plots of spiked tumor cell numbers from 0 to 100 are magnified for easier viewing (logarithmic axes).

The efficacy of the assay was determined through spike-in experiments. Tumor cells were defined from fluorescent images according to the criteria DAPI+ and CK+ and CD45–, while the white blood cells were defined as DAPI+ and CK–and CD45+. A typical example of captured images of tumor cells and white blood cells in the spike-in experiments is shown in Fig 2A. Bright-field images were also considered as a criterion, as shown in Fig 2B. For evaluation of linearity with respect to spiked cancer cell number, we conducted the experiments using three number sets of spiked cells: around 10 cells (8–13 cells, actually counted before being spiked-in), around 100 cells (80–114 cells, actually counted before being spiked-in), and around 1000 cells (approximate number of cells). As shown in Fig 3, a plot of the number of detected cells against the number of spiked cells of SK-BR-3 and PC-9 showed linearity, with regression line slopes of 0.7885 and 0.8189, and determination coefficients (*R* ^2^) of 0.9809 and 0.9999, respectively. The mean percentages (and CVs) of detected cells (S2 Table) were: SK-BR-3: 90.0% (12.5%) for ~10 cells, 70.6% (5.0%) for ~100 cells; and PC-9: 70.2% (18.4%) for ~10 cells, 85.2% (2.7%) for ~100 cells. PC-14, H69 and SBC-3, the cell lines displaying relatively low CK expression (as confirmed in Figure a in S2A Fig), showed a relatively small number of detected cells (with mean percentages of 13.9%, 6.4%, and 3.0% for ~100 cells, respectively), which was presumably caused by weakness in the immunofluorescent signal of the CK with a combination of the FITC-conjugated anti-CK mAb and the Alexa Fluor 488-conjugated anti Pan-CK mAb AE1/AE3, although the combination of these antibodies covers the majority of the CK isoforms (S2B Fig). As evidence of this, application of another immunofluorescent staining protocol, utilizing the anti-mouse IgG_1_ secondary antibody, aiming to amplify the signal of the primary CK antibody, as well as Alexa Fluor 488 dye as a brighter chromophore than FITC, showed relatively high percentages (and CVs) of detected cells: PC14: 63.3% (26.8%) for ~10 cells, 68.6% (24.7%) for ~100 cells; H69: 70.1% (10.9%) for ~10 cells, 72.7% (10.5%) for ~100 cells; and SBC-3: 50.2% (8.4%) for ~10 cells, 55.9% (7.5%) for ~100 cells; whereas the percentages of detected cells did not significantly differ from those obtained when using FITC-labeled primary anti-CK mAb for SK-BR-3 and PC-9 (data not shown). In the case of H69 and SBC-3, both small cell lung cancer cell lines, the relatively small cell radius may account for the relatively low detection rate. As described above, the DEP force exerted on cells of relatively smaller size (the average diameter of H69, for example, is 12.5 μm) [21] is less than on cells of relatively larger size (the diameter range of SK-BR-3 is about 12–30 μm) [35], which may account for the difference in detection rate for these small-cell lines in the study. If so, the low detection rate for relatively small tumor cells might be theoretically overcome by increasing the AC voltage amplitude and/or length of application, to enhance the DEP force effect. These results suggest that our negative enrichment method has robustness, and that the amplification of a weak CK signal due to weak expression of CK, by means of a secondary antibody conjugated to a brighter chromophore, is useful for detecting CTCs with various characteristics.

Next, head-to-head comparison with the CellSearch system was performed in a blinded manner; that is, testing without notifying examiners of the species and numbers of cells spiked into the blood. Each sample in our system was prepared by spiking an arbitrary number of tumor cells of any one species from SK-BR-3, PC-9, PC-14, H69 or SBC-3 into 3 mL of blood. In parallel, for comparison with the CellSearch system, blood samples into which tumor cells had been spiked with a number of cells proportional to blood volume (10 mL) were prepared in a CellSave collection tube, and 7.5 mL of the blood samples were analyzed using the CellSearch system. Samples not spiked with tumor cells were also analyzed using both systems. The results are summarized in Table 1. The mean percentage of detected cells (our system vs. the CellSearch system) were: for EpCAM/CK-high SK-BR-3 (*n* = 5), 86.6% vs. 69.5%; for EpCAM/CK-high PC-9 (*n* = 5), 82.7% vs. 91.2%; for EpCAM-null/CK-low PC-14 (*n* = 4), 51.5% vs. 0.0%; for EpCAM/CK-low H69 (*n* = 5), 65.6% vs. 50.5%; and for EpCAM-null/CK-low SBC-3 (*n* = 4), 51.0% vs. 0.2%. Notably, when spiking a low number of cancer cells into blood (2–4 cells into 3 mL of blood), certainty of cancer cell detection (4/4) was confirmed. In such cases, our system lost as few as 1 or 2 cells during serial procedures involving enrichment, entrapment, and immunofluorescent staining. Using our system, one cell was detected as a tumor cell in a few 3-mL healthy donor samples (2/5), while the CellSearch system also detected one or two cells as tumor cells, in a few 7.5-mL healthy donor samples (2/5), indicating a similar threshold number of CTCs for determining whether a patient is CTC positive or negative, in the case of both systems. These results suggest that our CTC detection system is advantageous, in comparison with the CellSearch system, for detecting EpCAM-null cell lines, displaying equal efficacy in detecting EpCAM/CK high cell lines.

**Table 1:** Comparative Detection Data from the Spike-in Experiments using Tumor Cell Lines (our System vs. the CellSearch System).

	Our system					CellSearch system					
Cell Line	Number of spiked cells / 3 mL blood	Number of detected cells	Detection rate	Mean Detection Rate	CV	Number of spiked cells		Number of detected cells	Detection rate	Mean Detection Rate	CV
						/ 10 mL blood	/ 7.5 mL blood				
SK-BR-3 (n = 6)	3	2	66.7%	-	-	10	8	5	66.7%	-	-
	4	3	75.0%	-	-	14	11	7	66.7%	-	-
	9	9	100.0%	86.6%	14.1%	33	25	18	72.7%	69.5%	6.7%
	55	46	83.6%			183	137	88	64.1%		
	105	80	76.2%			350	263	188	71.6%		
	1000	767	76.7%	-	-	3333	2500	3077	123.1%		
PC-9 (n = 5)	2	1	50.0%	-	-	9	7	5	74.1%	-	-
	5	5	100.0%	-	-	14	11	7	66.7%	-	-
	10	8	80.0%	82.7%	-	29	22	22	101.1%	91.2%	-
	89	76	85.4%		-	346	260	211	81.3%		-
	1000	1420	142.0%	-	-	3333	2500	2567	102.7%	-	-
PC-14 (n = 4)	13	6	46.2%	51.5%	12.5%	40	30	0	0.0%	0.0%	-
	24	13	54.2%			74	56	0	0.0%		
	134	62	46.3%			446	335	0	0.0%		
	113	67	59.3%			360	270	0	0.0%		
H69 (n = 5)	10	7	70.0%	65.6%	29.2%	36	27	13	48.1%	50.5%	16.2%
	19	9	47.4%			67	50	28	55.7%		
	29	22	75.9%			99	74	37	49.8%		
	98	88	89.8%			330	248	149	60.2%		
	149	67	45.0%			496	372	144	38.7%		
SBC-3 (n = 4)	16	8	50.0%	51.0%	6.5%	54	41	0	0.0%	0.2%	-
	67	37	55.2%			223	167	1	0.6%		
	93	44	47.3%			363	272	0	0.0%		
	103	53	51.5%			343	257	1	0.4%		
Healthy donor (n = 5)	-	0	-	-	-	-	-	0	-	-	-
	-	0	-	-	-	-	-	0	-	-	-
	-	1	-	-	-	-	-	0	-	-	-
	-	1	-	-	-	-	-	1	-	-	-
	-	0	-	-	-	-	-	2	-	-	-

Footnote: CV: coefficient of variation.

### Genomic analysis of single tumor cells

**Fig 4:**
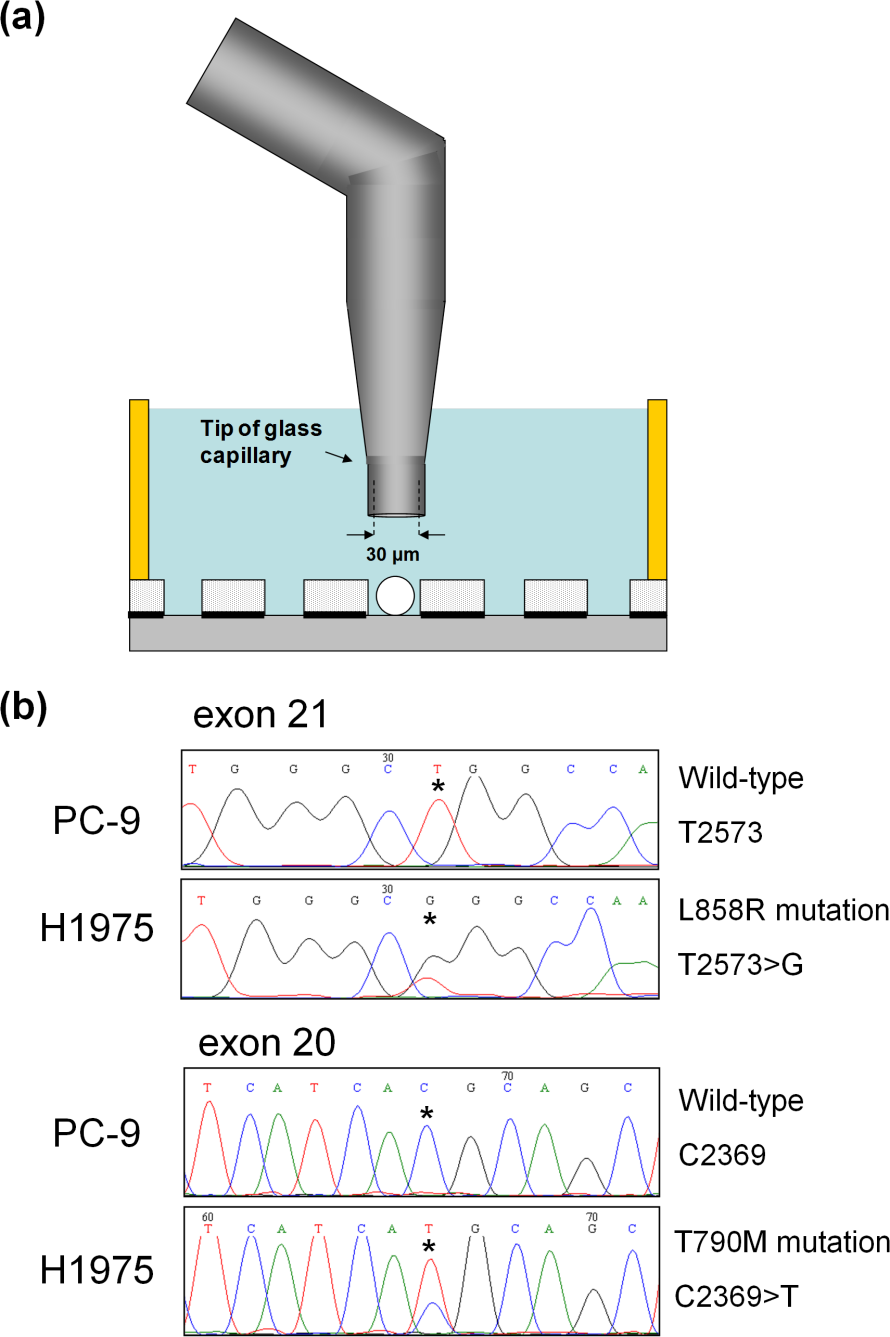
(a) Schematic drawing of single tumor cell isolation. Targeted cells are aspirated with an L-shaped glass capillary, and released into a PCR tube. **(b)** Sequencing chromatograms with T790M *EGFR* exon 20 mutation and L858R *EGFR* exon 21 mutation, obtained from WGA product from a single H1975 cell isolated by our CTC detection system. The NSCLC cell line H1975, which harbors a T790M mutation on exon 20 and an L858R mutation on exon 21 of the *EGFR*, were spiked into blood, followed by serial procedures. Isolated cells were separately subjected to WGA, followed by Sanger direct sequencing. The asterisks in the chromatograms indicate the positions of both mutations: at nucleotide 2369 (C→T), which leads to substitution of methionine for threonine at position 790; and at nucleotide 2573 (T→G), which leads to substitution of arginine for leucine at position 858. Both mutations were successfully detected. In contrast, when the NSCLC cell line PC-9 with neither mutation was used, neither mutation was detected.

To enable mutation analysis of CTCs isolated by the microwell array after immunofluorescent staining, the entrapped tumor cell samples were retrieved with an L-shaped glass capillary attached to a micromanipulator (Fig 4A). As shown in S3 Fig, we confirmed that in our system only targeted tumor cells were isolated, without aspiration of white blood cells in neighboring microwells. Isolated single tumor cells were then analyzed for the presence of specific mutations in each cell line using WGA, followed by Sanger direct sequencing. It is known that, in the case of NSCLC, a number of mutations in the *EGFR* are correlated with the efficacy of the molecularly-targeted drug Gefitinib (Iressa) (AstraZeneca, London, UK), which is targeting the EGFR molecule [36]. With the aim of obtaining a patient’s genomic profile from CTCs, we performed Sanger direct sequencing to detect a number of mutations in the *EGFR* of single tumor cells which had been isolated from the microwell array. For the experiment, we used the NSCLC cell line H1975, which harbors a T790M mutation on exon 20 and an L858R mutation on exon 21 of the *EGFR*, and the PC-9 cell line, which harbors neither mutation, as a negative reference. These cell lines were separately spiked into blood from a healthy donor, followed by entrapment, permiabilization, fixation and immunofluorescent staining as described above. To isolate single tumor cells by micromanipulated aspiration, the upper electrode substrate of the cell entrapment chamber was removed. Isolated cells were separately subjected to WGA, followed by Sanger direct sequencing. The primers used for Sanger direct sequencing are listed on S1 Table. Using WGA, a high success rate (80%, 12/15) was observed for entrapped single H1975 cells. The cases of failed WGA may be due to nucleus staining by DAPI which possibly damages DNA and/or the influence of the fixative reagent, suggesting that further optimization is needed in order to more fully characterize rare cells. As shown in Fig 4B and S5 Fig, as a result of Sanger direct sequencing of exon 20 and exon 21, using only successful WGA product from a single H1975 cell as a template, sequence chromatograms with L858R mutation (T2573>G) and T790M mutation (C2369>T) were obtained. In contrast, sequence chromatograms of exon 21 and exon 20 with neither mutation were obtained from WGA product from a single PC-9 cell. These results demonstrated that single tumor cells isolated by our CTC detection system are durable for further genomic analysis.

## Discussion

### Advantages of our dielectrophoretic methods for entrapping cells in the microwell array

We have confirmed that almost all targeted cells were entrapped in the microwell array after the cell entrapment procedure utilizing dielectrophoresis (DEP) (S4 Fig). There are several methods for entrapping cells in microarrays, including sedimentation by gravity [37], centrifugation [38], the filter-based microcavity array technique [21], or a combination of immunomagnetic selection and filter-based purification using a single microchip [39]. In terms of time of entrapment and amount of cell loss, our system utilizing DEP force is able to entrap up to 3 ×10^5^ cells per single microarray chamber, with no cell loss, in significantly less time (3 min) than the gravity method (15 min) [37]. Further, in the case of both the gravity and centrifugation methods, cells settled on the substrate surface outside the wells will inevitably be lost, due to the lack of a drawing force, such as the DEP force, directing cells into the wells (Ref. 37 and S4 Fig). Although the methods utilizing size-based enrichment are also able to enrich and entrap cells in less time, they risk losing small and/or deformable cells while the size and deformability of the CTCs has yet to be revealed [35]. The combined immunomagnetic selection and filter-based purification method runs a similar risk, though enabling efficient collection of entrapped cells for further molecular examination [39]. In addition to saving time, the continuous application of DEP force in our system minimizes loss of cells due to detachment from the microwells during medium exchange to the poly-L-lysine solution, which enhances overall cell attachment to the microwells. After attachment of cells to the microwells, several types of solution including organic solvent (e.g., ethanol for permeabilization), and cell staining solution (e.g., fluorophore-conjugated antibody, or cell staining reagent such as hematoxylin or eosin), are applicable to ensure maximal cell retention. Given that lowering the concentration of the formaldehyde added to the cell-permiabilizing ethanol solution had a weakening effect on the strength of cell attachment to the microwells, observable in decreased cell retention during the cell attachment procedure using poly-L-lysine solution and the subsequent immunofluorescent staining procedure (data not shown), the formaldehyde may possibly contribute to the strength of cell attachment and fixation, through cross-linkage between the lysine residues in the poly-L-lysine molecule and the cell surface proteins. Our optimized cell entrapment, attachment, and immunofluorescent staining protocol in this study resulted in both low cell loss (< 5% of entrapped cells) and robust isolation of single cells by aspiration.

### Singulation of cells in the microwell array

For reliable analysis of rare cells such as CTCs, it is preferable that there be no contamination from undesired cells such as white blood cells. Our DEP system for cell entrapment, however, is unable to entrap single cells in individual microwells in a controlled fashion. Specifically, if the cell suspension loaded into the cell entrapment chamber contains an equal or larger number of cells than the number of microwells in the chamber, multiple cells could, in principle, be trapped in the same microwell. To overcome this problem, we reduced the number of cells in a given suspension to be loaded into the cell entrapment chamber, using the negative enrichment method. In this study, the cell entrapment chambers possessed approximately 300,000 microwells, and two cell entrapment chambers were used for one blood sample. In this case, the cell number in the suspension should be reduced to below 600,000. In future clinical studies, the number of white blood cells would naturally vary between patients, and therefore the cell number and requisite number of cell entrapment chambers should both be determined before loading the cell suspension into the chamber(s). In addition, in spite of our reduction in the cell number, it occasionally happened that more than one cell was entrapped in a given microwell, as shown in Fig 2B and S3 Fig As the number of white blood cells per microwell was dependent on the total number of cells introduced into the chamber, the larger the total number of cells, the more frequently such multiple entrapment occurred (S6 Fig). Thus, in our system, a single cancer cell and a few white blood cells may occasionally be harvested together for further molecular analysis. In such cases, the important factors are the ratio of the amount of DNA yielded from a given cancer cell, and the detection sensitivity of the molecular analysis, based on the performance of the WGA kit used in our study, which can amplify the DNA content from samples containing greater numbers of cells (up to 1000). The typical values of detection sensitivity for mutational analysis are 25% for Sanger sequencing, 5–10% for next-generation sequencing associated with whole genome amplification, and 1–5% for qPCR. With regard to sensitivity, a single cancer cell with up to 4 white blood cells in a single microwell is sufficient for further molecular analysis.

### Comparison with other systems utilizing dielectrophoresis

Several systems utilizing dielectrophoresis for the separation or manipulation of cells have been applied to CTC analysis; among them, the dielectrophoretic field-flow-fractionation (DEP-FFF) system [29, 30] and the Di-Electro-Phoretic Array system (DEPArray system) [27, 28]. The DEP-FFF system is a powerful tool for molecular marker-independent separation of tumor cells from blood. However, for further characterization of tumor cells separated using the DEP-FFF system, an additional procedure involving plating on a slide glass is required for immunofluorescent staining [30], or some kind of cell isolating system (such as a cell sorter) or micromanipulation for single-cell genetic analysis. The DEPArray system, on the other hand, is a semiautomated system enabling the isolation of rare cells from mixed-cell populations at the single-cell level [27, 28]. However, as the DEPArray system requires reduction of the total number of cells to 10,000, considerable enrichment of the target cells is needed for single cell isolation by the system [27, 28]. Compared to these systems, our system enables seamless isolation of single cells through the enrichment process, and through the immunofluorescent labeling and washing process, as well as robust single-cell aspiration of the cells of interest from aligned microwells.

## Conclusions

We have provided the first report on the performance of our high-density dielectrophoretic microwell array system, a novel rare-cell capture system that enables the characterization and isolation of low numbers of cells from human blood samples at the single-cell level. We have described the analytical characteristics of the system, and provided proof-of-principle demonstrating its feasibility in the capture and molecular characterization of low numbers of tumor cells. The system has the following attractive features: (i) it is able to capture typical living human cells in individual microwells within a few minutes, with no bias such as surface markers, (ii) it is able to seamlessly isolate single cells from the enrichment process, (iii) the immunofluorescent labeling and washing process is performed on the cells retained in the microwell array, (iv) it enables robust single-cell isolation of the cells of interest from aligned microwells, and (v) it is compatible with all forms of downstream molecular analysis such as mutation detection. Ongoing improvement in whole-blood enrichment processing, and increasing the number of microwells, may further enhance the methodology by reducing microwell contamination from normal blood cells and increasing capture efficiency. The findings of this study would appear to suggest significant potential for its microwell array cell capture system, and further evaluation, using clinical samples, should be conducted.

## Supporting Information

S1 Fig Schematic Diagram of the Photolithographic Method, with Corresponding Etching, to Fabricate the Microwell Array Substrate. A Cr film was sputtered on the ITO surface of the substrate. Then, SU-8 negative-type photoresist was spin coated on the Cr surface. After this, the photoresist was subjected to UV exposure, using a photomask with a microwell pattern of microwell diameter 30 μm, followed by treatment with a developing solution. Exposure and developing times were adjusted such that the depth of the pores was 40 μm, which was equal to the film thickness of the photoresist. After development, the exposed Cr film was exfoliated with 30% ceric ammonium nitrate solution, exposing the ITO on the bottom surface of the microwells.
(PDF)

S2 Fig Scatter Plots of Immunofluorescent Analysis of Epithelial Marker Proteins in Cancer Cell Lines. The expression levels of epithelial adhesion molecule (EpCAM) and cytokeratin (CK) were assessed for each tumor cell line used in the spike-in experiments (**a**). SK-BR-3 and PC-9 showed a high expression level of both EpCAM and CK, suggesting epithelial property; whereas PC-14, H69 and SBC-3 showed a low expression level of CK and almost no expression of EpCAM. Isoforms of cytokeratin and detection properties of antibodies used in this study (**b**).
(PDF)

S3 Fig Isolation of Targeted Single Tumor Cells by Aspiration. SK-BR-3 cells were spiked into blood from a healthy donor, followed by entrapment, permiabilization, fixation, immunofluorescent staining, and single cell isolation, as described in the Material and Methods section. Successful aspiration of targeted single tumor cells (dotted circles), and no detachment of white blood cells in neighboring microwells, were confirmed.
(PDF)

S4 Fig Entrapment Rate of Tumor Cells with Various Frequencies. Cell entrapment analysis was performed to optimize the frequency of AC voltage applied between the pair of electrodes, for efficient entrapment of cells. After application of AC voltage with various frequencies for 3 minutes, the entrapment rate of live cells (stained with calcein AM) and dead cells (treated with 4% formaldehyde and stained with PI) was calculated, based on the number of live cells entrapped in microwells per the total number of live and dead cells in the region of interest.
(PDF)

S5 Fig Sequencing Chromatograms with T790M *EGFR* exon 20 Mutation and L858R *EGFR* exon 21 Mutation Obtained from WGA Product from 12 Single H1975 cells Isolated by our CTC Detection System. The NSCLC cell line H1975, which harbors a T790M mutation on exon 20 and an L858R mutation on exon 21 of the *EGFR*, were spiked into blood, followed by serial procedures. A total of 15 isolated single cells were separately subjected to WGA, and 12 successful WGA products were analyzed by Sanger direct sequencing. Arrows indicate the mutation positions: at nucleotide 2369 (C→T), which leads to substitution of methionine for threonine at position 790; and at nucleotide 2573 (T→G) which leads to substitution of arginine for leucine at position 858. The 12 WGA products were confirmed as identical with respect to the L858R region on exon 21. With respect to the T790M region on exon 20, 11 WGA products were confirmed as identical, while one product failed to be sequenced.
(PDF)

S6 Fig Number of White Blood Cells per Microwell as a Percentage of the Total Number of Microwells. 100k, 200k, and 270k white blood cells were introduced into a cell entrapment chamber possessing approximately 300,000 microwells. The number of white blood cells per microwell was dependent on the total number of cells introduced into the cell entrapment chamber: the larger the total number of cells, the more frequent the incidence of multiple cells in a single microwell.
(PDF)

S1 Table Primers Used for Sanger Direct Sequencing. (PDF)

S2 Table Results of Spike-in Experiments. (PDF)

S1 Text Supplemental Methods. (PDF)

S1 Video Demonstration of Cell Entrapment in the Microwell Array Utilizing Dielectrophoresis (DEP). A mixture of separately prepared white blood cells and SK-BR-3 cells (in the video, the SK-BR-3 cells are larger than the white blood cells) were introduced into the microwell substrate and settled, before the commencement of the video. Note that light-shielding Cr film was omitted from the substrate used in the supplemental video, for easy observation of cell locomotion caused by the DEP force. After commencing continuous application of AC voltage (at 5 s in the video), cells begin to be drawn toward nearby microwells by the DEP force. At 40 s in the video, almost all the cells have been entrapped in the microwells.
(MP4)
