# Supplementary material for: High-Density Dielectrophoretic Microwell Array for Detection, Capture, and Single-Cell Analysis of Rare Tumor Cells in Peripheral Blood
Source: PLoS One. 2015 Jun 24;10(6):e0130418. doi: 10.1371/journal.pone.0130418 (PMC4480363; doi:10.1371/journal.pone.0130418)
Supplement: S1 Fig — A Cr film was sputtered on the ITO surface of the substrate. Then, SU-8 negative-type photoresist was spin coated on the Cr surface. After this, the photoresist was subjected to UV exposure, using a photomask with a microwell pattern of microwell diameter 30 μm, followed by treatment with a developing solution. Exposure and developing times were adjusted such that the depth of the pores was 40 μm, which was equal to the film thickness of the photoresist. After development, the exposed Cr film was exfoliated with 30% ceric ammonium nitrate solution, exposing the ITO on the bottom surface of the microwells. (PDF) [file pone.0130418.s001.pdf]

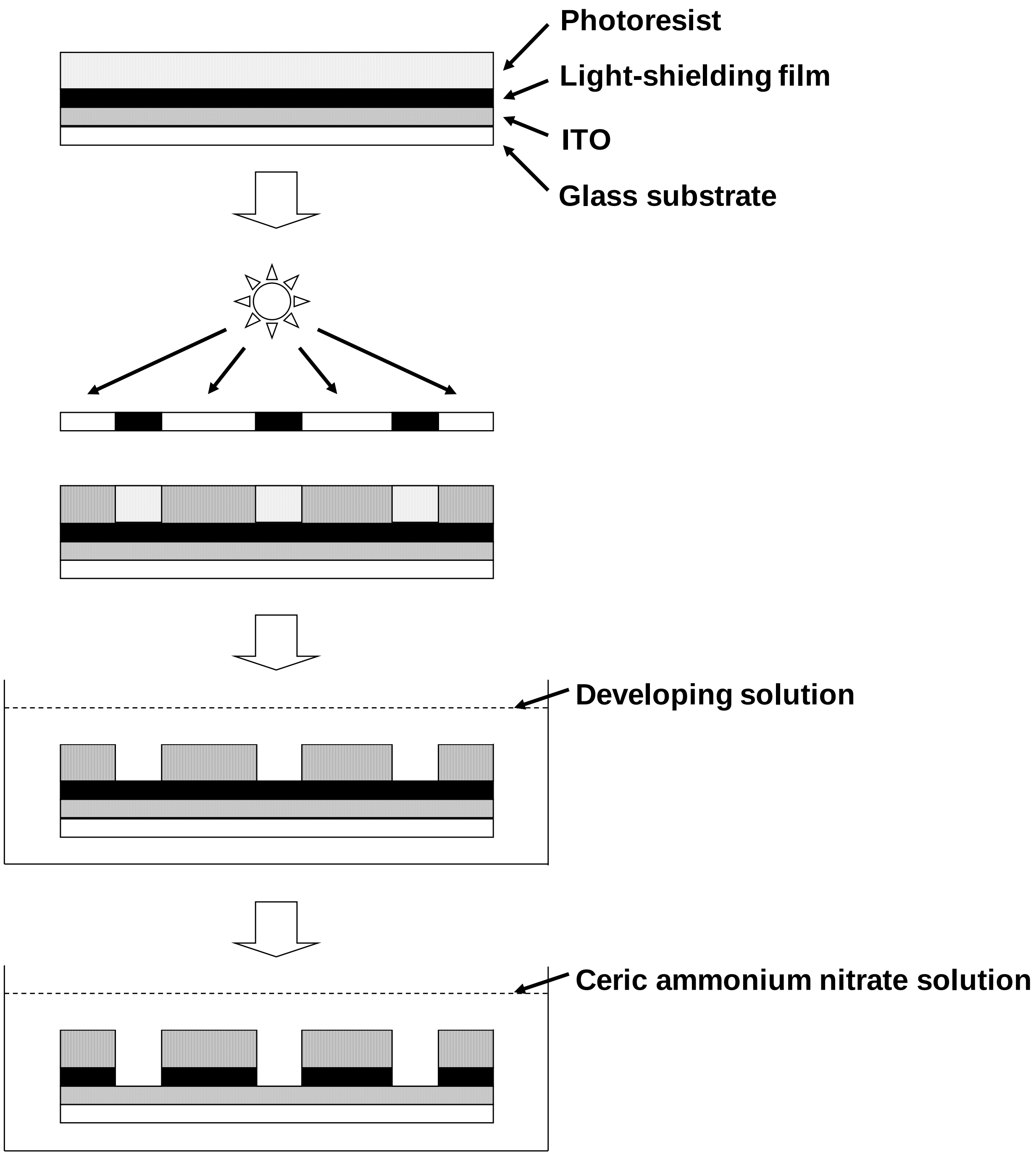

S1 Fig. Schematic Diagram of the Photolithographic Method, with Corresponding Etching, to Fabricate the Microwell Array Substrate.
