# Supplementary material for: High-Density Dielectrophoretic Microwell Array for Detection, Capture, and Single-Cell Analysis of Rare Tumor Cells in Peripheral Blood
Source: PLoS One. 2015 Jun 24;10(6):e0130418. doi: 10.1371/journal.pone.0130418 (PMC4480363; doi:10.1371/journal.pone.0130418)
Supplement: S2 Fig — The expression levels of epithelial adhesion molecule (EpCAM) and cytokeratin (CK) were assessed for each tumor cell line used in the spike-in experiments (a). SK-BR-3 and PC-9 showed a high expression level of both EpCAM and CK, suggesting epithelial property; whereas PC-14, H69 and SBC-3 showed a low expression level of CK and almost no expression of EpCAM. Isoforms of cytokeratin and detection properties of antibodies used in this study (b). (PDF) [file pone.0130418.s002.pdf]

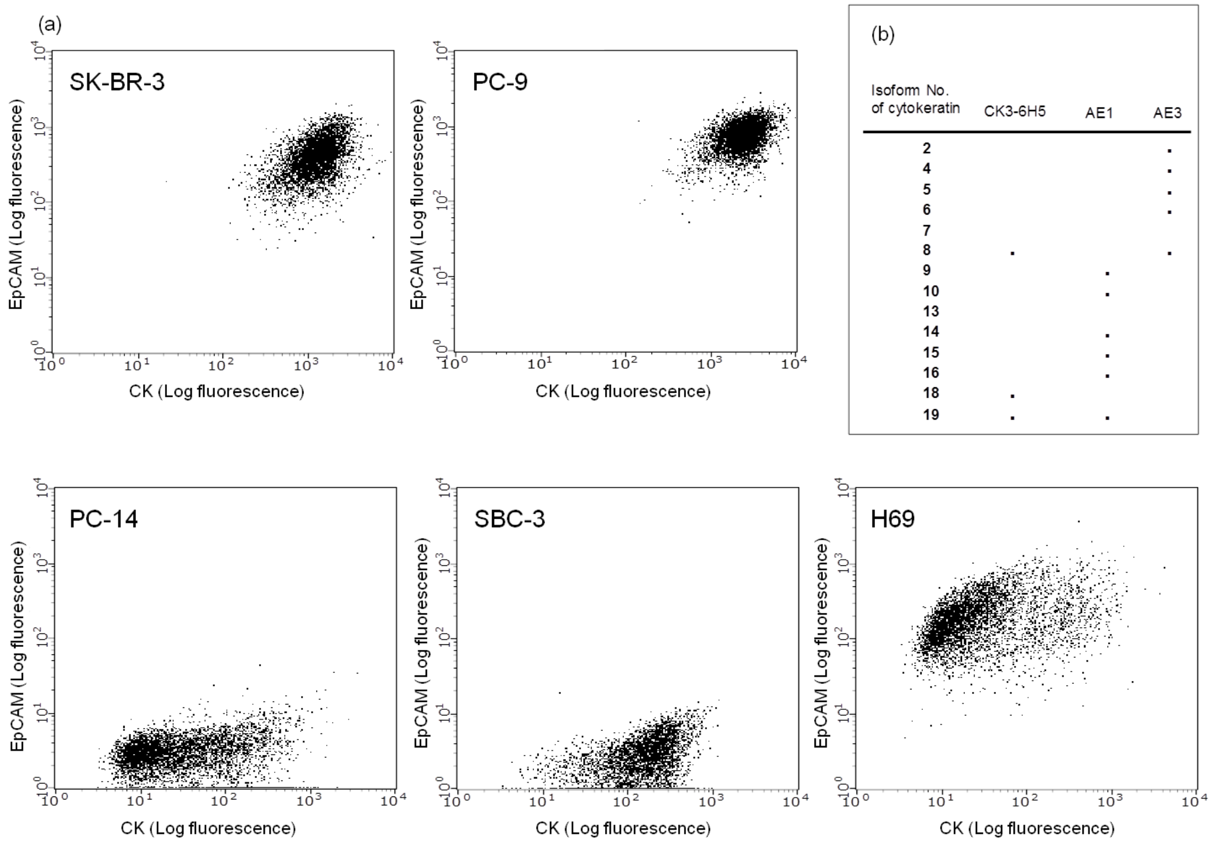

Isoform No. of cytokeratin	CK3-6H5	AE1	AE3
2			•
4			•
5			•
6			•
7			
8	•		•
9		•	
10		•	
13			
14		•	
15		•	
16		•	
18	•		
19	•	•	

S2 Fig. Scatter Plots of Immunofluorescent Analysis of Epithelial Marker Proteins in Cancer Cell Lines.
