# Supplementary figures and images for: High-Density Dielectrophoretic Microwell Array for Detection, Capture, and Single-Cell Analysis of Rare Tumor Cells in Peripheral Blood
Source: PLoS One. 2015 Jun 24;10(6):e0130418. doi: 10.1371/journal.pone.0130418 (PMC4480363; doi:10.1371/journal.pone.0130418)

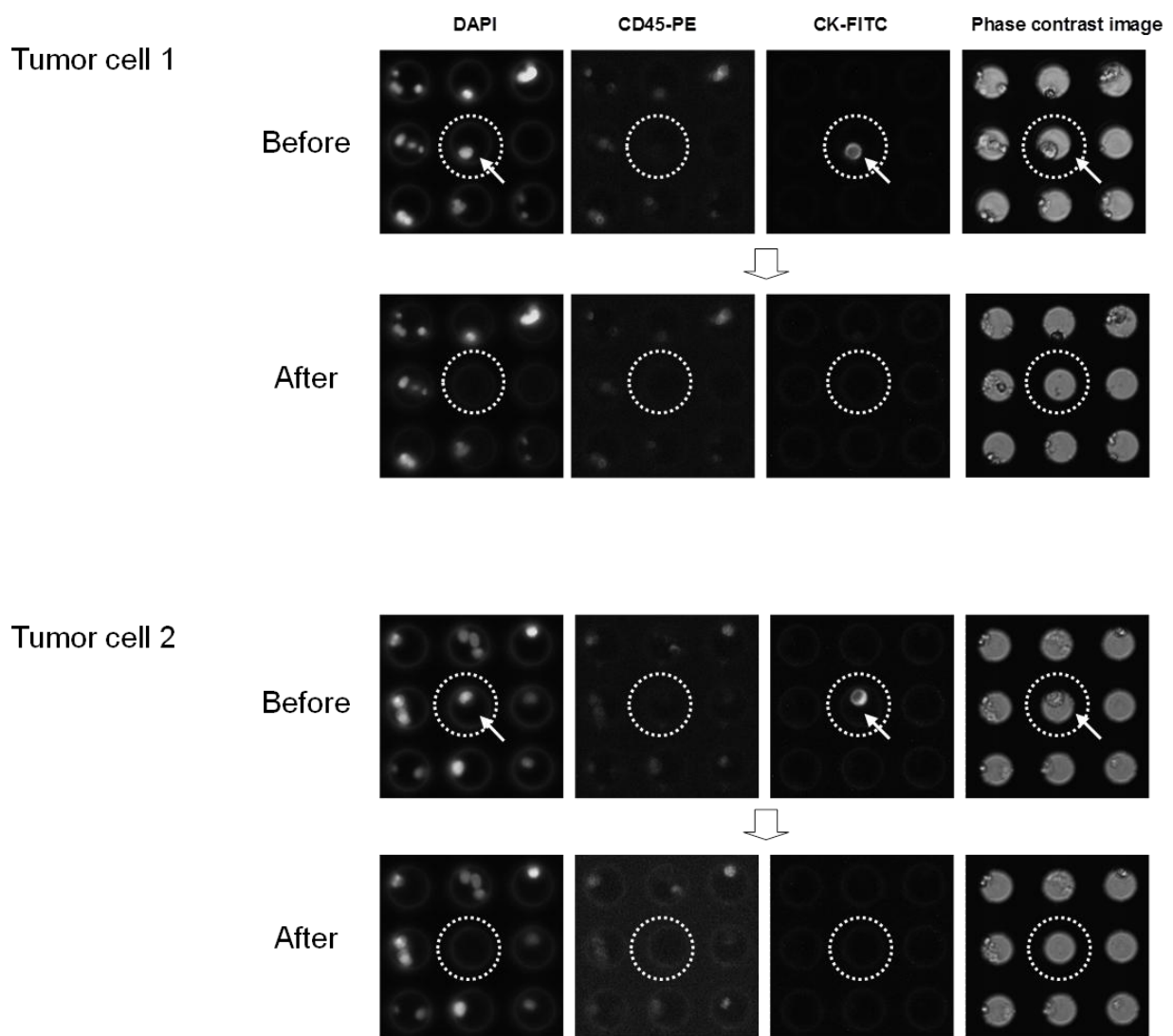

**S3 Fig. Isolation of Targeted Single Tumor Cells by Aspiration.**

Supplement: S3 Fig — SK-BR-3 cells were spiked into blood from a healthy donor, followed by entrapment, permiabilization, fixation, immunofluorescent staining, and single cell isolation, as described in the Material and Methods section. Successful aspiration of targeted single tumor cells (dotted circles), and no detachment of white blood cells in neighboring microwells, were confirmed. (PDF) [file pone.0130418.s003.pdf]

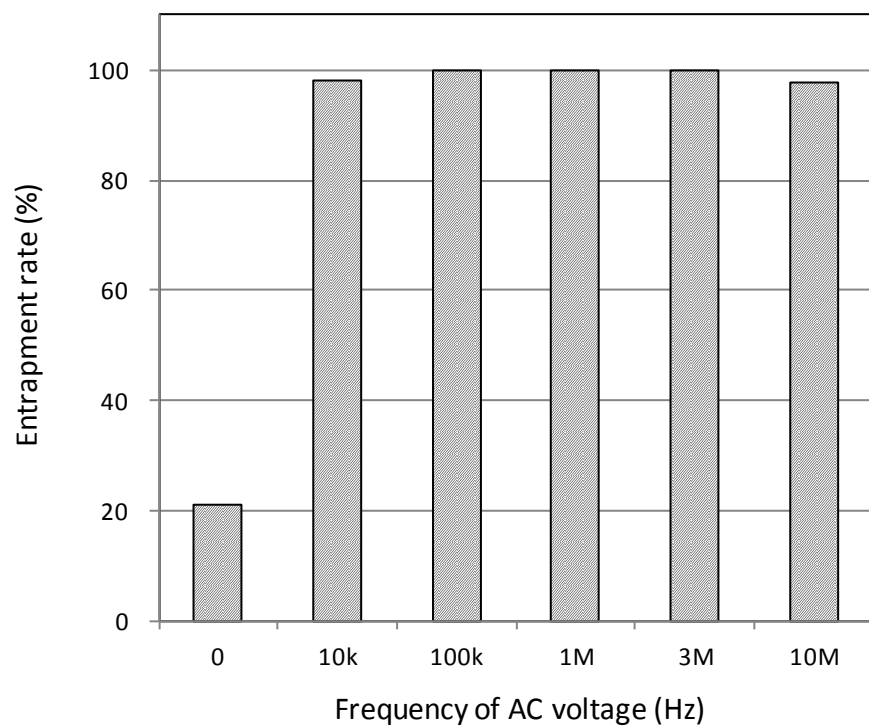

**S4 Fig. Entrapment Rate of Tumor Cells with Various Frequencies.**

Supplement: S4 Fig — Cell entrapment analysis was performed to optimize the frequency of AC voltage applied between the pair of electrodes, for efficient entrapment of cells. After application of AC voltage with various frequencies for 3 minutes, the entrapment rate of live cells (stained with calcein AM) and dead cells (treated with 4% formaldehyde and stained with PI) was calculated, based on the number of live cells entrapped in microwells per the total number of live and dead cells in the region of interest. (PDF) [file pone.0130418.s004.pdf]
