# Supplementary material for: High-Density Dielectrophoretic Microwell Array for Detection, Capture, and Single-Cell Analysis of Rare Tumor Cells in Peripheral Blood
Source: PLoS One. 2015 Jun 24;10(6):e0130418. doi: 10.1371/journal.pone.0130418 (PMC4480363; doi:10.1371/journal.pone.0130418)
Supplement: S5 Fig — The NSCLC cell line H1975, which harbors a T790M mutation on exon 20 and an L858R mutation on exon 21 of the EGFR, were spiked into blood, followed by serial procedures. A total of 15 isolated single cells were separately subjected to WGA, and 12 successful WGA products were analyzed by Sanger direct sequencing. Arrows indicate the mutation positions: at nucleotide 2369 (C→T), which leads to substitution of methionine for threonine at position 790; and at nucleotide 2573 (T→G) which leads to substitution of arginine for leucine at position 858. The 12 WGA products were confirmed as identical with respect to the L858R region on exon 21. With respect to the T790M region on exon 20, 11 WGA products were confirmed as identical, while one product failed to be sequenced. (PDF) [file pone.0130418.s005.pdf]

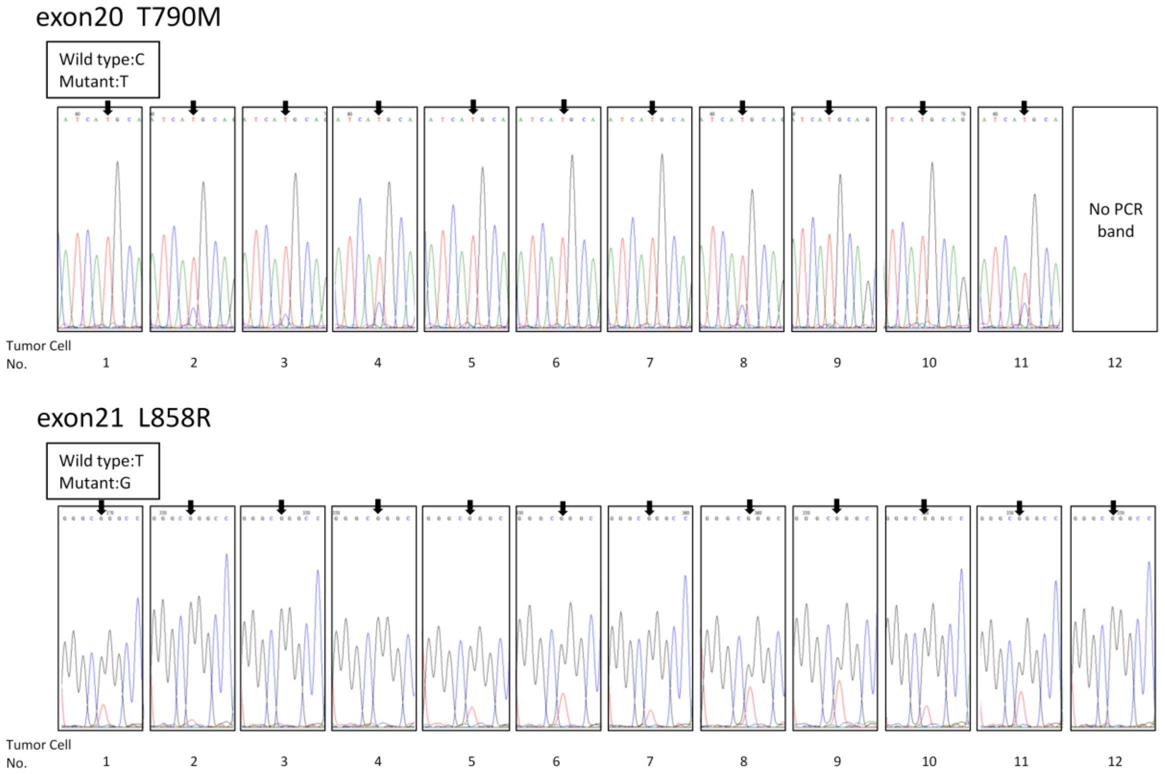

S5 Fig. Sequencing Chromatograms with T790M *EGFR* exon 20 Mutation and L858R *EGFR* exon 21 Mutation Obtained from WGA Product from 12 Single H1975 cells Isolated by our CTC Detection System.
