# Supplementary material for: High-Density Dielectrophoretic Microwell Array for Detection, Capture, and Single-Cell Analysis of Rare Tumor Cells in Peripheral Blood
Source: PLoS One. 2015 Jun 24;10(6):e0130418. doi: 10.1371/journal.pone.0130418 (PMC4480363; doi:10.1371/journal.pone.0130418)
Supplement: S6 Fig — 100k, 200k, and 270k white blood cells were introduced into a cell entrapment chamber possessing approximately 300,000 microwells. The number of white blood cells per microwell was dependent on the total number of cells introduced into the cell entrapment chamber: the larger the total number of cells, the more frequent the incidence of multiple cells in a single microwell. (PDF) [file pone.0130418.s006.pdf]

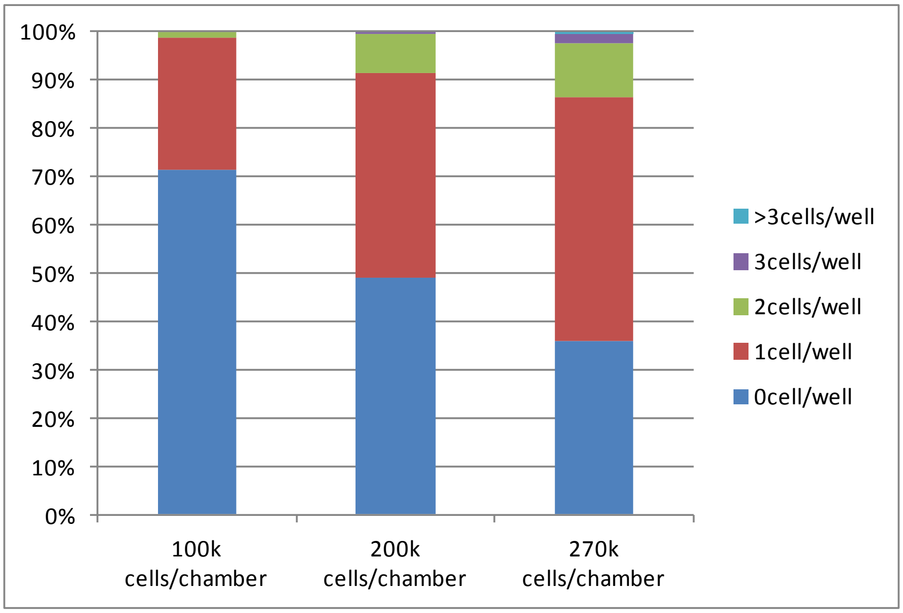

S6 Fig. Number of White Blood Cells per Microwell as a Percentage of the Total Number of Microwells.
