# Supplementary material for: High-Density Dielectrophoretic Microwell Array for Detection, Capture, and Single-Cell Analysis of Rare Tumor Cells in Peripheral Blood
Source: PLoS One. 2015 Jun 24;10(6):e0130418. doi: 10.1371/journal.pone.0130418 (PMC4480363; doi:10.1371/journal.pone.0130418)
Supplement: S1 Table — (PDF) [file pone.0130418.s007.pdf]

S1 Table. Primers Used for Sanger Direct Sequencing.

GENE	No. of Exon	Primer type	Sequence of primers
EGFR	20	PCR primers	Forward: 5′-cacactgacgtgcctctcc-3′<br>Reverse: 5′-ccgtatctcccttccctgat-3′
		Sequencing primer	5′-ctccctccaggaagcctacgtgat-3′
	21	PCR primers	Forward: 5′-cctcacagcagggtcttctc-3′<br>Reverse: 5′-ggaaaatgctggctgaccta-3′
		Sequencing primer	5′-agccaggaacgtactggtga-3′
