# Supplementary material for: High-Density Dielectrophoretic Microwell Array for Detection, Capture, and Single-Cell Analysis of Rare Tumor Cells in Peripheral Blood
Source: PLoS One. 2015 Jun 24;10(6):e0130418. doi: 10.1371/journal.pone.0130418 (PMC4480363; doi:10.1371/journal.pone.0130418)
Supplement: S2 Table — (PDF) [file pone.0130418.s008.pdf]

S2 Table. Results of Spike-in Experiments.

Cell line	Cell number set	Number of spiked cells / 3 mL blood	Number of detected cells	Detection rate (%)	Mean detection rate (%)	CV (%)
SK-BR-3	10 cells*	9	7	77.8	90.0	12.5
		13	12	92.3		
		9	9	100.0		
	100 cells*	96	78	81.3	70.6	20.2
		80	61	76.3		
		103	56	54.4		
	1000 cells*	1000	693	69.3		
		1000	897	89.7		
		1000	767	76.7		
PC-9	10 cells*	12	9	75.0	70.2	18.4
		10	8	80.0		
		9	5	55.6		
	100 cells*	103	90	87.4	85.2	2.7
		99	82	82.8		
		89	76	85.4		
	1000 cells*	1000	824	82.4		
		1000	815	81.5		
		1000	1420	142.0		
PC-14	10 cells#	11	7	63.6	63.3	26.8
		10	8	80.0		
		13	6	46.2		
	100 cells*	106	17	16.0	13.9	
		111	13	11.7		
	100 cells#	105	75	71.4	68.6	24.7
		100	75	75.0		
		113	67	59.3		
	1000 cells#	1000	1053	100.0		
		1000	763	76.3		
H69	10 cells#	9	7	77.8	70.1	10.9
		8	5	62.5		
		10	7	70.0		
	100 cells*	96	3	3.1	6.4	
		103	10	9.7		
	100 cells#	103	69	67.0	72.7	10.5
		114	70	61.4		
		98	88	89.8		
	1000 cells#	1000	889	88.9		
		1000	616	61.6		
		1000	242	24.2		
SBC-3	10 cells#	11	6	54.5	50.2	8.4
		13	6	46.2		
		10	5	50.0		
	100 cells*	91	0	0.0	3.0	
		101	6	5.9		
	100 cells#	98	57	58.2	55.9	7.5
		93	58	62.4		
		93	44	47.3		
	1000 cells#	1000	535	53.5		
		1000	544	54.4		

Immunofluorescent protocol utilizing:
* : fluorophore-conjugated primary anti-CK antibody
# : primary anti-CK antibody and fluorophore-conjugated secondary antibody
