# Supplementary material for: High-Density Dielectrophoretic Microwell Array for Detection, Capture, and Single-Cell Analysis of Rare Tumor Cells in Peripheral Blood
Source: PLoS One. 2015 Jun 24;10(6):e0130418. doi: 10.1371/journal.pone.0130418 (PMC4480363; doi:10.1371/journal.pone.0130418)
Supplement: S1 Text — (PDF) [file pone.0130418.s009.pdf]

S1 Text. Supplemental Methods.

Cell Culture. The breast cancer cell line SK-BR-3 and the non-small cell lung cancer (NSCLC) cell line PC-9 were obtained from DS Pharma Biomedical (Osaka, Japan) and Immuno-Biological Laboratories (Fujioka, Japan), respectively. The NSCLC cell line H1975 was obtained from American Type Culture Collection (ATCC) (Manassas, VA). The NSCLC cell line PC-14 and the small cell lung cancer (SCC) cell line H69 were kindly provided by Dr. F. Koizumi (National Cancer Center, Tokyo, Japan).The NSCLC cell line PC-14 was provided by Professor Y. Hayata, Tokyo Medical College (Tokyo, Japan). The small-cell lung cancer (SCC) cell line H69 was established at the National Cancer Institute, Bethesda, MD, and was obtained from Dr. Y. Shimosato, National Cancer Center Hospital. The SCC cell line SBC-3 was obtained from the Health Sciences Research Resource Bank (Osaka, Japan). The SK-BR-3 cells were cultured in McCoy's 5a (GIBCO, Life Technologies, Grand Island, NY) containing 10% fetal bovine serum (FBS, GIBCO). The PC-9 cells were cultured in D-MEM/Ham's F-12 containing 10% FBS. The PC-14, H69, and SBC-3 cell lines were cultured in RPMI-1640 (Wako Pure Chemical Industries) containing 10% FBS. Cell lines were cultured under a humidified 5% CO_2/95% atmosphere at 37 ˚C. In the spike-in experiments, all the cell lines except H69 were harvested using 0.25% trypsin/1 mM EDTA (Wako Pure Chemical Industries), and then suspended in respective culture mediums. Since the H69 cell line showed floating aggregates morphology during culturing, the H69 cells were harvested and pipetted to form a discrete suspension before the experiments.

Fabrication of Microwell Array Substrate. A glass substrate coated with ITO was purchased from Photo Precision (Tokyo, Japan). A Cr film of 100 nm thickness was sputtered on the ITO

surface of the substrate. Then, a 40-μm layer of SU-8 negative-type photoresist (MicroChem, Newton, MA) was spin coated on the Cr surface. After natural drying for 1 minute, prebaking (95 ˚C, 3 minutes) was performed using a hot plate. After this, the photoresist was subjected to UV exposure, using a photomask with a microwell pattern of microwell diameter 30 μm and 50 μm longitudinal and latitudinal distance between pores, followed by treatment with a developing solution. Exposure and developing times were adjusted such that the depth of the pores was 40 μm, which was equal to the film thickness of the photoresist, and thus the Cr surface was exposed. After development, the exposed Cr film was exfoliated with 30% ceric ammonium nitrate solution, exposing the ITO on the bottom surface of the microwells. After this, postbaking (180 ˚C, 30 minutes) was performed using a hot plate, to cure the photoresist structure.

Negative Enrichment of Tumor Cells. After negative enrichment of the tumor cells performed using Rosettesep® reagent Human CD45 Depletion Cocktail, enriched mononucleated cells were harvested in a new 50 mL conical tube, and then washed with PBS containing 0.35% trisodium citrate. After centrifugation at 300 × g for 10 min at 25 ˚C, the supernatant was carefully discarded using a pipette, and then the pellet was suspended with the lysing solution and incubated for 5 min at room temperature (RT) for lysis of loosely associated red blood cells. After lysis, the suspension was diluted with 300 mM mannitol solution, followed by centrifugation at 300 × g for 5 min at 25 ˚C. The supernatant was discarded using a pipette, and then 300 mM mannitol solution was carefully added to the conical tube. After centrifugation at 300 × g for 5 min at 25 ˚C, the supernatant was discarded using a pipette.

Flowcytometric Analysis. Flowcytometric analysis was performed to assess the expression level of epithelial protein (EpCAM) and cytokeratin in the tumor cell lines used in the spike-in experiments. SK-BR-3, PC-9, PC-14, SBC-3, and H69 cell lines were harvested from the cell culture, and washed with PBS. Each cell line was then permeabilized with ethanol, supplemented with HCHO (formaldehyde), for 10 min in a test tube. After permeabilization, each tumor cell line was washed with PBS, and then incubated with PBS containing 1% BSA, 1:10 diluted phycoerythrin (PE)-conjugated anti-EpCAM mAb (mouse IgG_1) (Milenyi Biotec, Bergisch-Gladbach, Germany), 1:16 diluted CK-FITC (CK3-6H5), and 1 μg/mL of AE1/AE3-Alexa Fluor 488, for 10 min. After removal of unbounded antibodies, each cell line was washed with PBS, and suspended in PBS. Flowcytometric analysis was performed with a Guava EasyCyte™ flowcytometer (Merck KGaA, Darmstadt, Germany). The FITC, Alexa Fluor 488, and PE were excited by 488 nm blue laser, and fluorescence of the FITC and Alexa Fluor 488 was detected at 525 ± 15 nm, with fluorescence of PE detected at 583 ± 13 nm.

Cell entrapment analysis. Cell entrapment analysis was performed to optimize the frequency of AC voltage applied between the pair of electrodes, for efficient entrapment of cells. SK-BR-3 cell line was harvested in a test tube from cell culture, and washed with PBS. After 5 min incubation with carboxyfluorescein succinimidyl ester (CFSE) to label the live cells, the cells were washed with PBS, and then fixed with 4% formaldehyde for 10 min to maintain the cell membrane status. After being washed with 300 mM mannitol solution, the cells were incubated with propidium iodide (PI) to label cells with partially damaged membranes. After being washed with 300 mM mannitol solution, the cells were introduced into the cell entrapment chamber, followed by immediate application of 20 Vp-p AC voltage, with a square-wave shape and

various frequency, by a function generator (WF1974, NF Corporation, Tokyo, Japan), for 3 minutes. Then, the number of cells entrapped in microwells, and outside microwells, were counted using a BX 53 fluorescence microscope (Olympus, Tokyo, Japan). The entrapment rate of live cells was calculated based on the number of live cells entrapped in microwells per the total number of live cells in the region of interest.
